# Safety and Feasibility Study of a Novel Stent-Graft for Thoracic Endovascular Aortic Repair: a Canine Model Experiment

**DOI:** 10.21470/1678-9741-2016-0058

**Published:** 2017

**Authors:** Fan Yang, Jiehua Qiu, Zeliang Fu, Yuchen Qiu, Junrong Luo, Qingyang Xiao, Huabin Cao

**Affiliations:** 1 Institute of Animal Population Health, College of Animal Science and Technology, Jiangxi Agriculture University, Jiangxi, P.R. China.; 2 Department of Vascular Surgery, the Second Affiliated Hospital of Nanchang University, Jiangxi, P.R. China.; 3 APT Medical Inc., Hunan, P.R. China.

**Keywords:** Stent-graft, Thoracic Endovascular Aortic Repair, Dogs, Models, Animal, Safety, Feasibility Studies, Aortic Diseases, Endovascular Procedures, Stents

## Abstract

**Objective:**

To evaluate the safety and feasibility of a novel stent-graft for thoracic endovascular aortic repair (TEVAR) in a canine model, 9 adult hybrid dogs were used for the experiment.

**Methods:**

All animals were implanted with a novel thoracic aortic stent-graft via femoral artery. Blood sample was collected at pre-operation and 1, 2, 4, 8 and 12 weeks after implantation for hematological examination. Moreover, tissues from randomly selected 4 dogs were subjected to histopathological analysis with the optical microscope after stent-grafts were implanted for 3, 6, 9, and 12 months respectively. The experimental period lasted for more than 2 years.

**Results:**

A total of 9 stent-grafts were successfully implanted in the canine thoracic aortas and no migration or deformation occurred. Related indicators of blood routine, inflammatory factors, and immunology changes were not significantly (*P*>0.05), except the white blood cell (WBC) counts in the first week. Moreover, abnormal morphology was not found in all thoracic aortas via histopathological examination. Additionally, all stent-grafts were patent and did not migrate, and there was no thrombus in the lumens of stent-grafts.

**Conclusion:**

The novel thoracic aortic stent-graft made in China was safe and feasible for thoracic endovascular aortic repair in a canine model.

**Table:** 

Abbreviations, acronyms & symbols	
AAA	= Abdominal aortic aneurysm
AD	= Aortic dissection
C3	= Complement-3
CRP	= C-reactive protein
Hb	= Hemoglobin
H&E	= Hematoxylin and eosin
IgG	= Immunoglobulin G
IgM	= Immunoglobulin M
PLT	= Platelet
RBC	= Red blood cell
TAA	= Thoracic aortic aneurysm
TEVAR	= Thoracic endovascular aortic repair
TNF-α	= Tumor necrosis factor-α
WBC	= White blood cell

## INTRODUCTION

Aortic dissection (AD) is a life-threatening cardiovascular disease with high mortality^[1]^. Multiple abnormalities have been suggested to contribute to predisposition for this disease, mainly including hypertension, degeneration and genetic disorders^[2,3]^. The natural incidence of AD was 0.5-3.2/100.000/yr^[4,5]^, and there are about more than 10 thousand new cases of AD each year in China^[6]^. Meszaros et al.^[7]^ reported that about 50% patients died within 24 hours and 68.2% within the first 2 days after admission. Besides, the reporting cases of deaths due to aortic abnormalities including AD, thoracic aortic aneurysm (TAA) and abdominal aortic aneurysm (AAA) have accelerated more recently^[8]^.

Traditional surgical management technique for aortic diseases such as AD and TAA is surgical replacement with a prosthetic graft, whereas high postoperative complications and mortality have been described. Parodi et al.^[9]^ firstly reported that endovascular stent-graft treatment of AAA in 1991. Dake et al.^[10]^ used transluminally placed stent-grafts to treat descending TAA in 13 patients over a 24-month period. Endovascular stent-graft is capable of repairing various aortic abnormalities as well as maintaining blood flow and declining the possibility of aortic rupture. Therefore, thoracic endovascular aortic repair (TEVAR) by means of stent-graft has turned out to be a more attractive option than conventional surgical approaches^[11,12]^. However, TEVAR may result in kinking, endoleak, infolding or even stent-graft migration. Stent-graft disconformability and disattachment phenomena at the inner aortic arch radius in juvenile trauma victims have received widespread attention^[13,14]^. Although enormous applications have been found in China, most of stent-grafts for TEVAR come from import. Some Chinese patients cannot afford such high medical fees, and the specifications of the commercially available stent-grafts are unitary, which cannot satisfy the needs of complying with tapering naturally vessel, leading to high rates of long-term complication of endovascular stent-graft's distal end. Thus, it is urgent to independently develop a homegrown stent-graft with various tapers. This evaluation work was carried out to primarily evaluate the safety and feasibility of a novel thoracic aortic stent-graft designed by a company in a canine model.

## METHODS

### Compliance with Ethical Standards

All experimental procedures complied with the criteria in Guide for the Care and Use of Laboratory Animals and Brazilian Guidelines on Care and Use of Animals for Scientific and Teaching Purposes, National Council for Animal Experimentation Control. The institutional ethics committee also approved this study.

### Stent-graft System

**Fig. 1:**
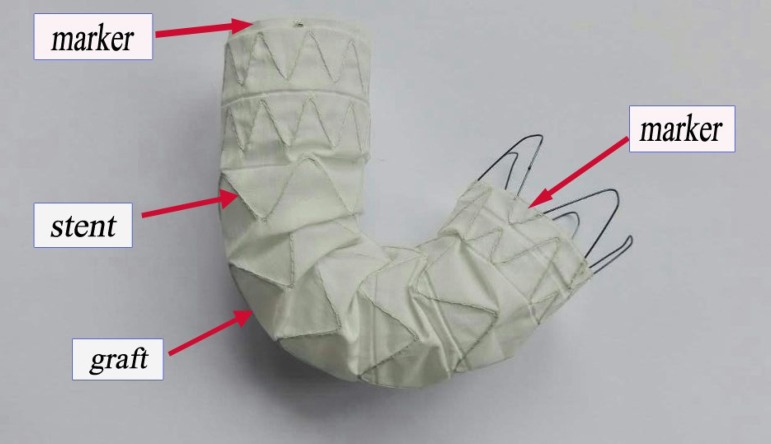
Stent-graft system

The stent-graft, 110 mm in length and 26 mm in diameter, is mainly comprised of nickel titanium alloy, polyethylene terephthalate and platinum-iridium alloy. It has characteristics such as good bendability, compliance, adhesion of vessel wall and low profile. The transportation system and delivery system are mainly composed of Teflon, polycarbonate and stainless steel, resulting in higher compliance. Moreover, the minimum size of transportation system is 14 F, while general products' sizes are 6-8 F. In addition, the external sheath of transportation system, merely 4.9 mm to 6.1 mm in diameter, can obviously reduce the damage of blood vessel. The markers on stent-graft are used to ensure accurate positioning (Figure 1).

### Animals and Treatments

The non-survival procedures were performed in compliance with national animal welfare laws. Nine adult hybrid dogs (weight 25 to 35 kg), used in this study, were derived from social donation after physical examination, vaccination and disinsectization performed. All animals were fed with special dog food and also guaranteed free access to water and provided an adequate housing. The dogs were handled and treated in accordance with strict guiding principles of the National Institutes of Health for experimental care and use of animals. The implantations were performed in a month and the experimental period lasted for more than 2 years. The order and time of necropsy are shown in Table 1.

**Table 1:** Order and time of necropsy of dogs.

Dog	1, 2	3, 4	5, 6	7, 8	9
Time of necropsy (after surgery)	3 months	6 months	9 months	12 months	> 12 months

### Operation

The operation was performed at Second Affiliated Hospital of Nanchang University. Dogs were given antibiotics for 3 days, 12 hours of fasting and water deprivation was carried out before surgery. The dogs were given a hypodermic injection of atropine sulfate (0.01 mg/kg) and etamsylate (10 mg/kg), respectively. Anesthesia was followed by intravenous propofol (6 mg/kg).

**Fig. 2:**
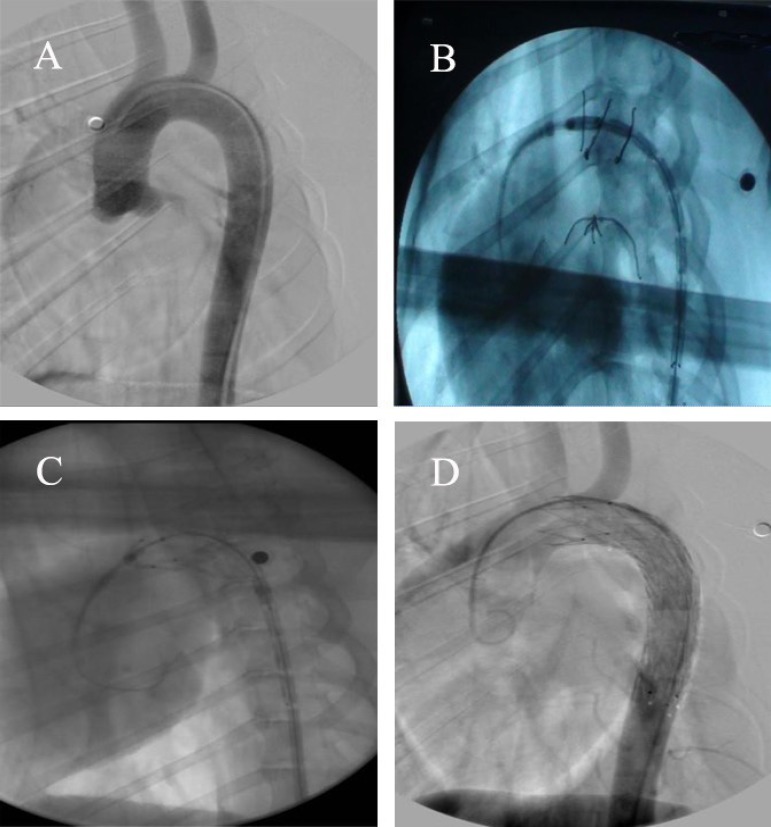
Angiography. (A) Preoperative angiography image. (B) The delivery system reached the present position. (C) The release of stent-graft firstly. (D) Postoperative angiography image.

The diameter of thoracic aorta was evaluated through the femoral artery. The heparin saline and contrast media were injected. And then, the loach guide wire and the pigtail catheter were inserted via common femoral artery. The distal end of the pigtail catheter should reach the position of ascending thoracic aorta. Angiography was conducted to measure the diameter of thoracic aorta (Figure 2). Afterwards, 260 cm in length with curved super-stiff guide wire were sent to the specific location of aortic arch via the pigtail catheter, and then the pigtail catheter and short needle sheath were extracted. Subsequently, the stent-graft system was sent to the desired position via the super-stiff guide wire. Finally, the stent-graft was unfolded and affixed to the vascular wall when it reached the desired position. Angiography was repeated to evaluate the effect of implantation (Figure 2).

### Blood Collection

Blood samples were collected from 9 dogs preoperatively and 1, 2, 4, 8 and 12 weeks after implantation. Two milliliters of blood collected via cephalic vein was used for the examination of blood routine. The remaining blood was allowed to clot, incubated at 37°C for 2 hours and the serum was obtained by centrifugation at 1000 g for 5 minutes at 4°C. Then the serum was stored at -20°C until analyzed for the examination of inflammatory factors and immunological indexes.

### Sacrifice and Tissue Collection

A complete necropsy was performed on 8 dogs at clinical veterinary medicine laboratory in the College of Animal Science and Technology Jiangxi Agricultural University. Histopathological examination was carried out on 4 dogs randomly selected 3, 6, 9 and 12 months after implantation. The thoracic aorta was dissected and washed with saline, then fixed in 10% neutral buffered formalin (3-5 days). Formalin-fixed samples were routinely processed, embedded in paraffin, sectioned at 5 µm, stained with hematoxylin and eosin (H&E), and pathological sections were observed using an optical microscope and photographs were taken.

### Statistical Analysis

The results were presented as mean ± SD. The statistical analysis included SPSS version 17.0 (SPSS Inc., Chicago, IL, USA), Student's t-test, GraphPad Prism 5.0 (GraphPad Inc., La Jolla, CA, USA). A *P* value of less than 0.05 was considered significant.

## RESULTS

No animal was killed during this experiment, except intentional sacrifice. Furthermore, no abnormality was found in habits and lifestyle on all animals before necropsy. The ninth animal was kept alive for late follow-up and it is showing good evolution.

### Results of the Blood Routine

**Fig. 3:**
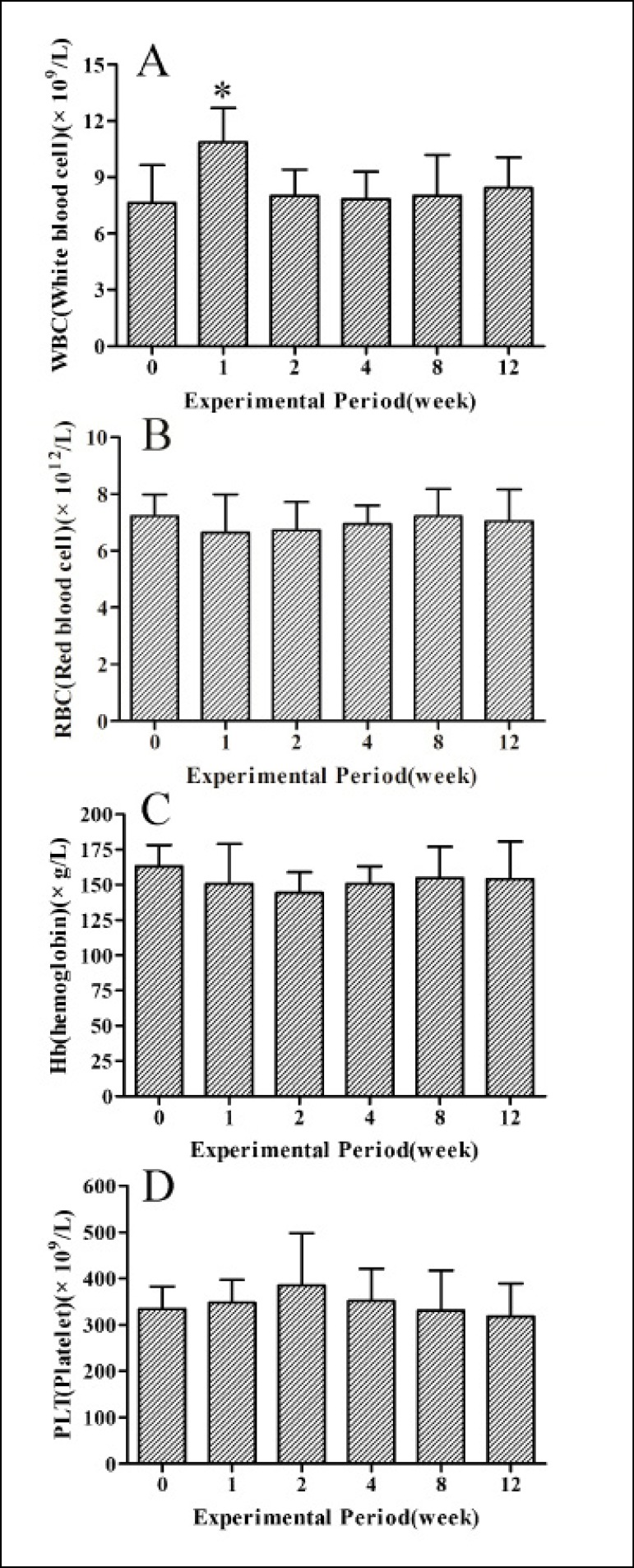
Blood routine indexes at pre- and 1, 2, 4, 8 and 12 weeks after implantation. Panels A-D show changes of WBC counts, RBC counts, Hb levels and PLT counts, respectively. *Donate significant difference (P<0.05). Data are the means ± SEM (n=9).

As shown in Figure 3, white blood cell (WBC) counts at the first week after implantation were significantly increased (*P*<0.05), in comparison with those at preoperative period, while there was no significant difference (*P*>0.05) among preoperative days and the other days (Figure 3A). Changes in red blood cell (RBC) counts, hemoglobin (Hb) levels and platelet (PLT) counts were not significant (*P*>0.05) among pre- and postoperative periods, and all indicators were kept in normal range in the experimental period (Figures 3B, C and D).

### Results of Inflammatory Factors

**Fig. 4:**
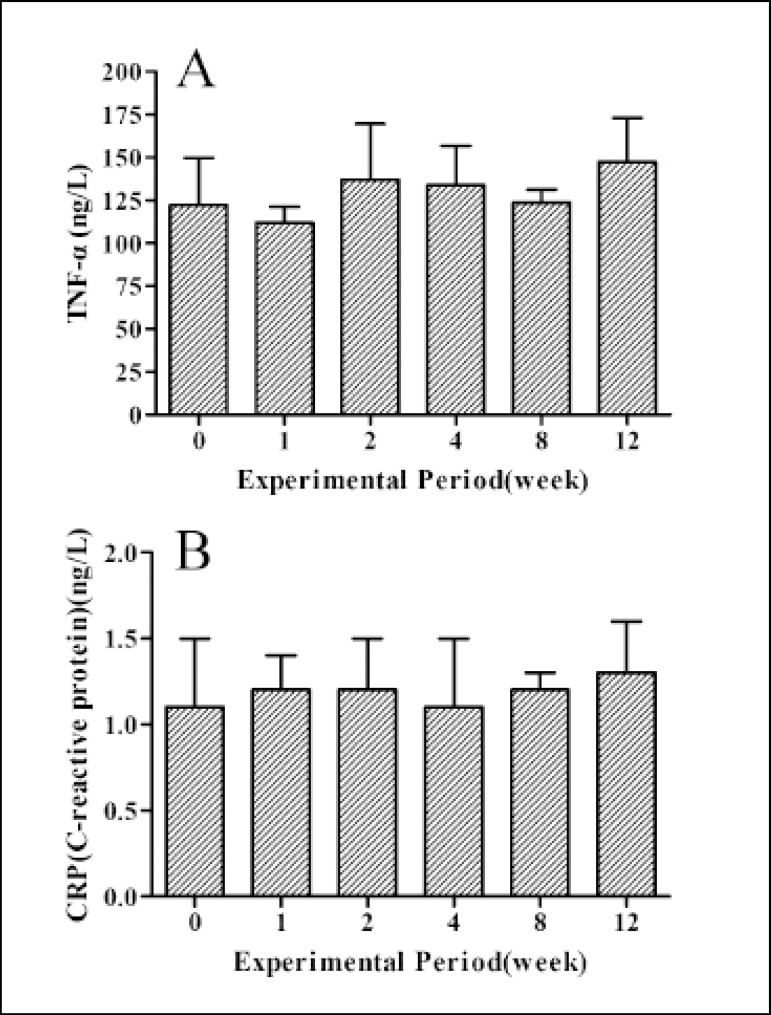
Inflammatory factors at pre- and1, 2, 4, 8 and 12 weeks after implantation. Panels A-B show changes of levels of TNF-α and CRP, respectively. Data are the means ± SEM (n=9).

As shown in Figure 4, the levels of tumor necrosis factor-α (TNF-α) (Figure 4A) and C-reactive protein (CRP) (Figure 4B) were kept in normal range during the experimental period and there was no significant difference (*P*>0.05) among pre- and postoperative periods.

### Results of Immunological Indexes

**Fig. 5:**
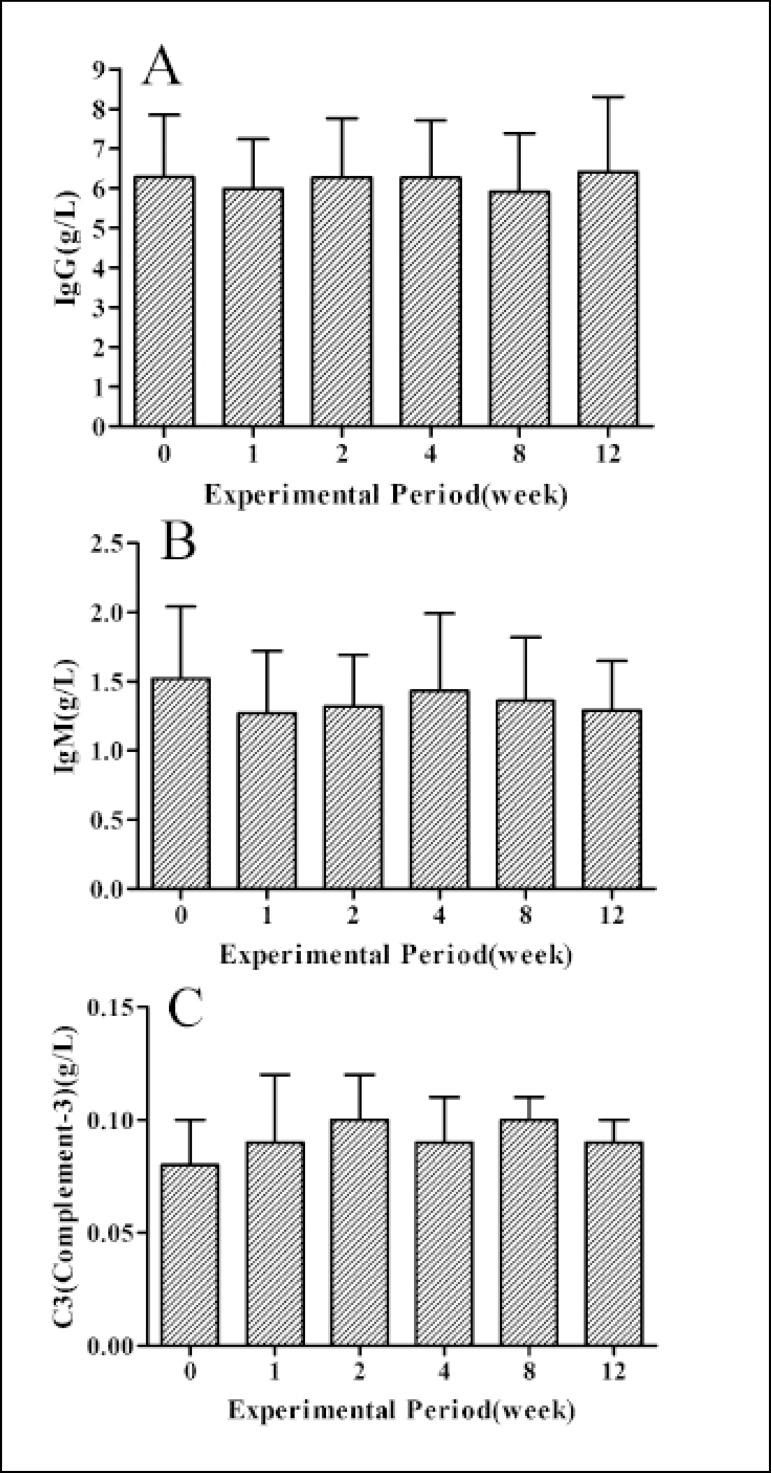
Immunological indexes at pre- and 1, 2, 4, 8 and 12 weeks after implantation. Panels A-C show changes of levels of C3, IgG and IgM, respectively. The data are the means ± SEM (n=9).

The changes of immunological indexes are shown in Figure 5. No significant difference (*P*>0.05) in the levels of complement-3 (C3), immunoglobulin G (IgG) and immunoglobulin M (IgM) among pre- and post-operative periods were observed and all indicators were kept in normal range (Figures 5A, B and C).

### Autopsy Findings

**Fig. 6:**
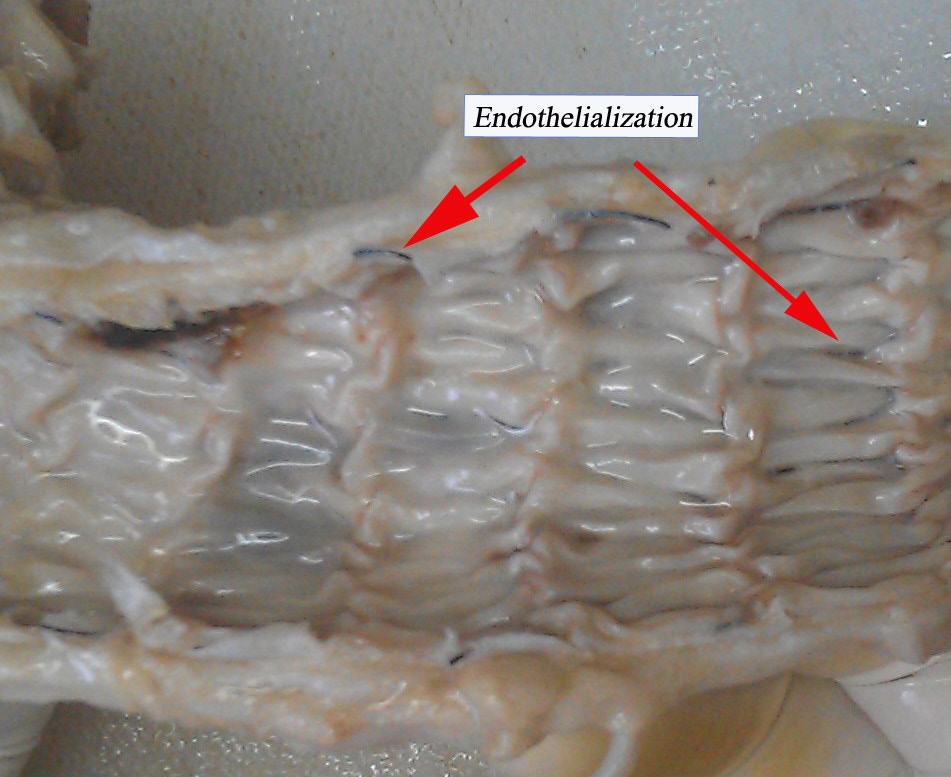
Thoracic aorta implanted with stent-graft.

All stent-grafts were patent and did not migrate. As shown in Figure 6, the inner surface of stent-graft was smooth, suggesting satisfactory neointimal coverage and the adventitia appeared essentially normal. Additionally, there was no thrombus in the lumens of stent-grafts. Endothelialization was found in the thoracic aorta implanted with stent-grafts, and perivascular erosion was not observed.

### Histopathological Examination

**Fig. 7:**
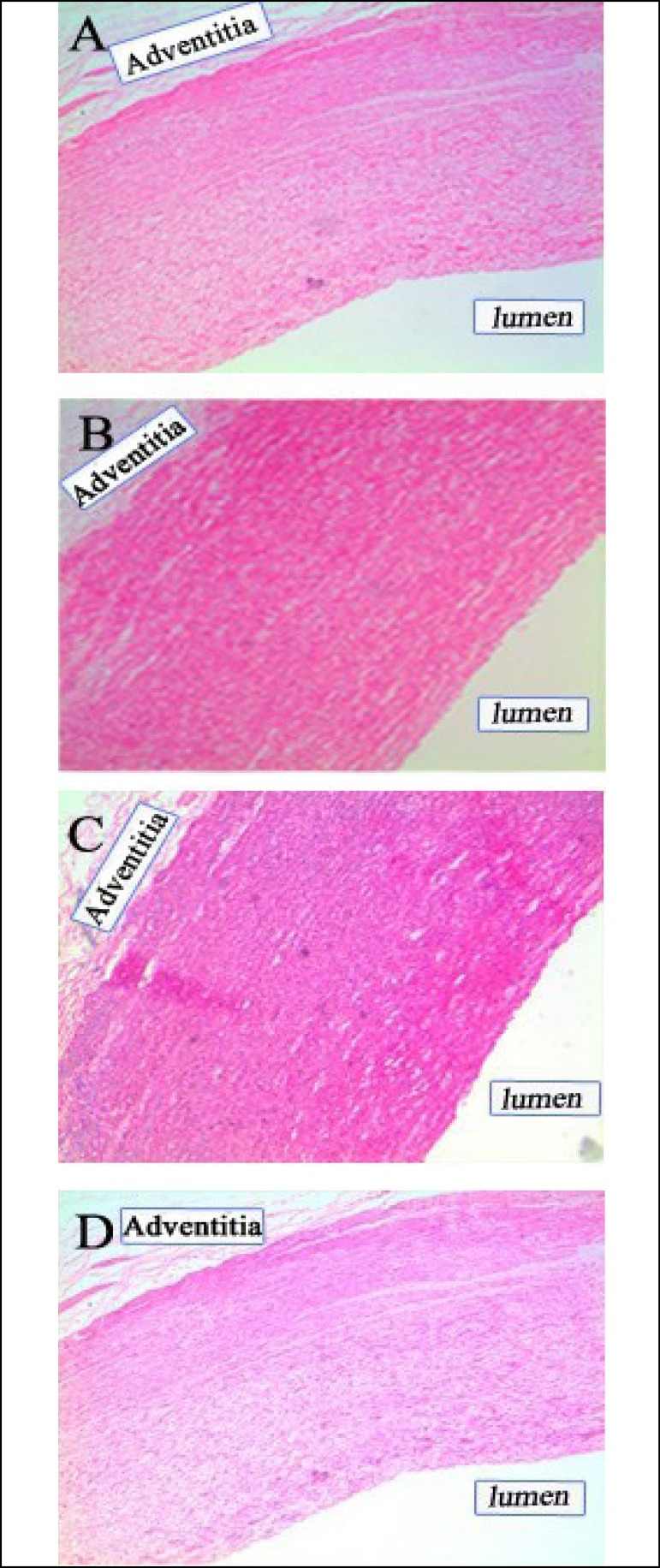
Histopathological changes in thoracic aorta with no stent-graft. (A) Thoracic aorta of 3 months after implantation. (B) Thoracic aorta of 6 months after implantation. (C) Thoracic aorta of 9 months after implantation. (D) Thoracic aorta of 12 months after implantation. Magnification, ×100.

**Fig. 8:**
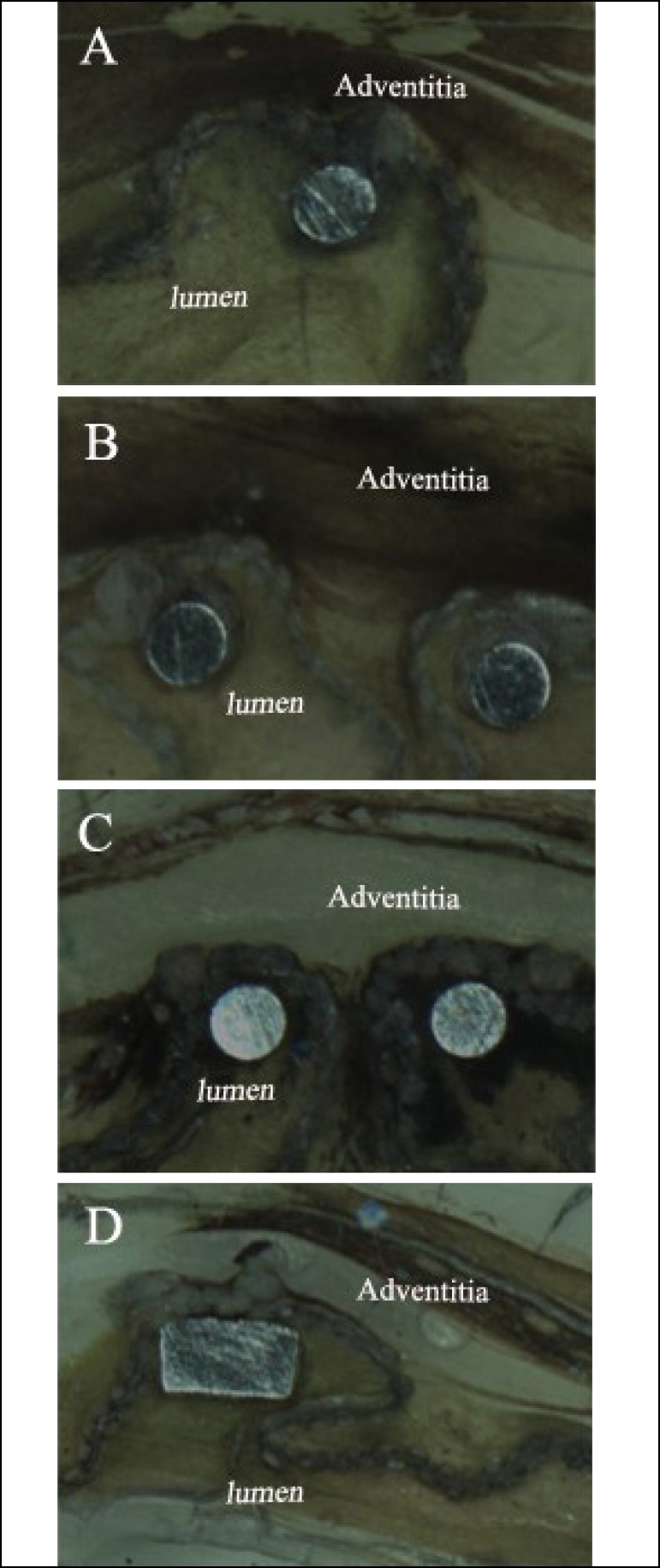
Histopathological changes in thoracic aorta implanted with stent-graft. (A) Thoracic aorta of 3 months after implantation. (B) Thoracic aorta of 6 months after implantation. (C) Thoracic aorta of 9 months after implantation. (D) Thoracic aorta of 12 months after implantation. Magnification, ×100.

The histopathological results of thoracic aorta with no stent-graft are shown in Figure 7. All the tissue slices showed normal morphology. No inflamed cells (mononuclear cell, macrophage, etc.) and degeneration or necrosis were found. Additionally, the histopathological results in the thoracic aorta implanted with stent-graft are shown in Figure 8. All the tissue slices showed no abnormal sign. Abnormal cells and thoracic aorta thickening were not observed.

## DISCUSSION

The application of endovascular repair by means of stent-graft has been demonstrated to be a safe and effective treatment for various diseases of thoracic aorta^[15,16]^. This technology has been widely applied in China. The demand for stent-graft is on the increase due to the huge population of patients in China who are suffering from aorta diseases. However, most of stent-grafts in China rely on imports now. Moreover, it is difficult to afford the medical fees for people because China is a developing country. Besides, the specifications of these stent-grafts are unitary, which cannot satisfy the needs of complying with tapering naturally vessel, leading to high rates of long-term complication of endovascular stent-graft's distal end. It is urgent to independently design an effective homegrown stent-graft, which has various tapers and can decline the rates of long-term complication of endovascular stent-graft's distal end. The stent-graft used in our study has good bendability, compliance, adhesion of vessel wall and low profile, and can obviously reduce the damage of blood vessel, which could solve the deficiencies in general products.

In this study, experimental animals were used to evaluate the safety and feasibility of the stent-graft because it was convenient for real-time observation. Dogs (weight 25 to 35 kg) were adopted in this research, as the diameter of their artery and its hemodynamics are approximate to human. Furthermore, their tolerance is strong enough to survive long hours of anesthesia and surgery. Canine fibrinolytic system of is more active than the human^[17]^, and their mortality rate and postoperative complications are much lower than other animals.

Aortic abnormalities such as AD and TAA are harmful diseases with high mortality in emergencies. Endovascular stent-grafts not only could repair various aortic abnormalities, but also maintain blood flow and decline the possibility of aortic rupture. However, there were plenty of problems of early first-generation stent-grafts, such as vascular injury, ascending aortic dissection or aortic penetration from struts, stroke with insertion, graft collapse, endovascular leaks, graft material failure, continued aneurysm expansion or rupture, and migration or kinking^[14]^. Besides, inflammation and rejection are also worth of concerning in complication. In this study, the obvious increase of WBC counts at the first week after the implantation, while there was no remarkable difference in other days, reflected inflammation caused by surgery, which could also be demonstrated by the results of inflammation factor. However, it had been improved significantly after using antibiotic. The results of immunological indexes revealed that rejection was not obvious *in vivo*. Some innovative studies on endovascular repair in animal model have been conducted to study aortic diseases, but they were short-term studies^[18,19]^. All dogs had stent-grafts implanted successfully in this study. Focus should be attached to the materials of stent-graft in implantation, as well as the importance of surgery itself. Multiple reasons have been confirmed to contribute to fail, mainly including anesthesia, intubation failure, hemorrhage, postoperative infection and improper nursing care.

In our study, the autopsy results showed that the stent-graft could fit the vessel wall of thoracic aorta well. The formation of intima on the inner surface of the bare stent and complete endothelialization of stent-graft surface were observed. The histopathological results also showed that neointima had formed and adventitia appeared essentially normal. The results demonstrated that the stent-graft had high histocompatibility and sealability and had not induced inflammation and calcification, which were consistent with majority relevant reports^[20,21]^. Additionally, the experimental period was more than 2 years, so that the long-term dynamic changes could be distinctly observed, which further demonstrated the safety of stent-graft.

In short, further research is still needed before final clinical application.

## CONCLUSION

In the present study, the thoracic aortic stent-graft made in China was safe and feasible for TEVAR in a canine model.

**Table:** 

Authors' roles & responsibilities	
FY	Design and draft of the work; final approval of the manuscript version to be published
JQ	Draft of the work and review it critically for important intellectual content; final approval of the manuscript version to be published
ZF	Contributions to the analysis and interpretation of data for the work; final approval of the manuscript version to be published
YQ	Contributions to the analysis and interpretation of data for the work; final approval of the manuscript version to be published
JL	Calibration; final approval of the manuscript version to be published
QX	Calibration; final approval of the manuscript version to be published
HC	Design and draft of the work; final approval of the manuscript version to be published
